# Supplementary material for: Glycaemic level and glycaemic variability in acute ischaemic stroke and functional outcome: An observational continuous glucose monitoring study
Source: PLoS One. 2025 May 30;20(5):e0318456. doi: 10.1371/journal.pone.0318456 (PMC12124563; doi:10.1371/journal.pone.0318456)

G-VAS

Glycaemic Variability in Acute Stroke

1.2 01/04/2020

MAIN SPONSOR: Imperial College London

FUNDERS: Dexcom

STUDY COORDINATION CENTRE: Imperial College London

IRAS Project ID: 279997

REC reference: xxx

**Protocol authorised by:**

| **Name & Role** | **Date** | **Signature** |
| --- | --- | --- |
|  |  |  |
|  |  |  |
|  |  |  |
|  |  |  |
|  |  |  |
|  |  |  |

**Study Management Group**

Chief Investigator: Dr Neil Hill

Co-investigators: Dr Soma Banerjee

Dr Omid Halse

Prof Nick Oliver

Statistician: Dr Ian Godsland

Study Management: Ms Maria Thomas

**Study Coordination Centre**

For general queries, supply of study documentation, and collection of data, please contact:

**Study Coordinator**: Maria Thomas

**Address**: Department of Metabolism, Digestion and Reproduction

Imperial College London,

Hammersmith Hospital, Commonwealth Building, Level 7, Room 7.S7

Du Cane Road, White city, London, W12 0NN

**Te**l: 020 7594 8995

**E-mail**: m.thomas@imperial.ac.uk

**Clinical Queries**

Clinical queries should be directed to Dr Neil Hill who will direct the query to the appropriate person

**Sponsor**

Imperial College London is the main research Sponsor for this study. For further information regarding the sponsorship conditions, please contact the Head of Regulatory Compliance at:

Joint Research Compliance Office

Imperial College London and Imperial College Healthcare NHS Trust

Room 215, Level 2, Medical School Building

Norfolk Place

London, W2 1PG

**Tel**: **0207 594 9459/ 0207 594 1862**

<http://www3.imperial.ac.uk/clinicalresearchgovernanceoffice>

**Funder**

Dexcom

This protocol describes the G-VAS study and provides information about procedures for entering participants. Every care was taken in its drafting, but corrections or amendments may be necessary. These will be circulated to investigators in the study. Problems relating to this study should be referred, in the first instance, to the Chief Investigator.

This study will adhere to the principles outlined in the UK Policy Frame Work for Health and Social Care Research. It will be conducted in compliance with the protocol, the Data Protection Act and other regulatory requirements as appropriate.

**Table of Contents Page No**

[1. INTRODUCTION 7](#_Toc32837804)

[1.1 Background 7](#_Toc32837805)

[1.2 Rationale for current study 9](#_Toc32837806)

[1.3 Future Work 9](#_Toc32837807)

[2. STUDY OBJECTIVES 10](#_Toc32837808)

[3. STUDY DESIGN 12](#_Toc32837809)

[4. Participant Entry 13](#_Toc32837810)

[4.1 Pre-registration evaluations 13](#_Toc32837811)

[4.2 Inclusion Criteria 13](#_Toc32837812)

[4.3 Exclusion Criteria 13](#_Toc32837813)

[4.4 Withdrawal criteria 13](#_Toc32837814)

[5. adverse events 14](#_Toc32837815)

[5.1 Definitions 14](#_Toc32837816)

[5.2 Reporting Procedures 14](#_Toc32837817)

[6. ASSESSMENT AND FOLLOW-UP 16](#_Toc32837818)

[6.1 Recruitment and consent 16](#_Toc32837819)

[6.2 Enrollment and study conduct 18](#_Toc32837820)

[6.3 Telephone follow up at 3 months 18](#_Toc32837821)

[7. STATISTICs and data analysis 19](#_Toc32837822)

[8. regulatory issues 20](#_Toc32837823)

[8.1 Ethics approval 20](#_Toc32837824)

[8.2 Consent 20](#_Toc32837825)

[8.3 Confidentiality 20](#_Toc32837826)

[8.4 Indemnity 20](#_Toc32837827)

[8.5 Sponsor 20](#_Toc32837828)

[8.6 Funding 20](#_Toc32837829)

[8.7 Audits 21](#_Toc32837830)

[9. Study Management 22](#_Toc32837831)

[11. References 24](#_Toc32837832)

[12. APPENDICES 26](#_Toc32837833)

[Appendix 1. Summary of investigations, treatment and assessments 26](#_Toc32837834)

[Appendix 2. Data Flow Mapping 27](#_Toc32837835)

**Glossary of Abbreviations**

| CGM | Continuous Glucose Monitoring |
| --- | --- |
| GV | Glycaemic Variability |
| SD | Standard Deviation |
| MAG | Mean Absolute Glucose |
| NIHSS | National Institutes of Health Stroke Score |
| MRS | Modified Rankin Scale |
|  |  |
|  |  |
|  |  |
|  |  |
|  |  |
|  |  |
|  |  |
|  |  |
|  |  |
|  |  |
|  |  |
|  |  |
|  |  |
|  |  |
|  |  |
|  |  |
|  |  |
|  |  |
|  |  |
|  |  |
|  |  |
|  |  |
|  |  |
|  |  |
|  |  |
|  |  |

**Keywords**

Acute ischaemic stroke, diabetes, glycaemic variability

**Study Summary**

| **TITLE** | Glycaemic Variability in Acute Stroke |
| --- | --- |
| **DESIGN** | Observational cohort study |
| **AIMS** | Establish the impact of glycaemic variability on outcomes in acute stroke |
| **OUTCOME MEASURES** | **Glycaemic Primary outcome:** Mean absolute glucose (MAG) change during initial 72 hours after confirmed stroke  **Stroke Primary outcomes:** National Institutes of Health Stroke Scale (NIHSS) and Modified Rankin Scale (MRS) |
| **POPULATION** | N=200 with or without diabetes in 1:1 ratio |
| **ELIGIBILITY** | Acute Stoke (NIHSS>6) with or without diabetes |
| **duration** | Duration of hospital stay up to a maximum of 3 months. 1 year total duration. |

# 1. INTRODUCTION

## Background

People with diabetes who have a stroke have worse outcomes (Burton JK et al. 2019; Muir KW et al. 2011; Masrur S et al. 2015). Evidence for tight glycaemic control (e.g. maintaining blood glucose between 4.0 and ~7.5mmol/L) on the days immediately after stroke is lacking; studies have not shown improved outcomes and have noted higher rates of hypoglycaemia in intensively treated patients (Bellolio MF et al. 2014; American Heart Association. 2019). However the National Institute for Health and Care Excellence Guidance states that, ‘People with acute stroke should be treated to maintain a blood glucose concentration between 4 and 11mmol/L and hyperglycaemia (determined by both admission blood glucose and HbA1c) is associated with adverse outcomes (National Institute for Health and Care Excellence*.* 2017). In practice, maintaining blood glucose levels below 12mmol/L in people with acute stroke can be challenging, in particular when parental feeding is required.

Intermittent glucose measurement and measures of protein glycation provide limited information on the dynamic changes in glucose over time and do not take into account variability in glucose concentrations. Glycaemic variability (GV) is the consequence of multiple endogenous and exogenous factors and is a measurable variable.

To measure GV a data series of glucose values is required. These may be derived from continuous glucose monitoring and may be from within one time period (such as a day) or over several periods, allowing comparisons between periods. Initial methodologies for GV calculation were defined for self-monitoring data and newer methodologies have been expressly designed for continuous monitoring data.

There is no minimum length of time defined for satisfactory glycaemic variability calculation but, as with all statistical measures, the larger the dataset the more robust the metrics. Glucose concentration is not normally distributed about the mean. There is a long ‘tail’ to the glucose distribution extending into the hyperglycaemic range. Measures such as standard deviation do not take into account this asymmetric distribution and are thus relatively insensitive to hypoglycaemia. Hypoglycaemia is a significant barrier to improving glycaemic control and is a source of anxiety to people with diabetes. Not only that, it is unpleasant, is associated with morbidity and mortality and contributes to the global healthcare and financial burden of diabetes.

In vitro data has suggested that GV is more deleterious than consistent hyperglycaemia. Human umbilical vein endothelial cells exposed to a glucose concentration alternating between 5 and 20mmol/L every 24 hours show significantly more apoptosis than cells exposed to a constant concentration of 5mmol/L or 20mmol/L over 14 days (Risso A, et al. 2001). Using the same constant and alternating glucose concentrations in human umbilical vein endothelial cells overproduction of reactive oxygen species is highest with oscillating glucose concentrations (Quagliaro L, et al. 2003). In the same sequence of studies expression of the cytokine IL-6 was highest with oscillating glucose concentrations (Piconi L, et al. 2004).

In human proximal tubular cells exposed to increased glucose concentrations (25mmol/L), cell growth, collagen synthesis and cytokine production are elevated, and this is increased further by oscillating the glucose concentration between 25mmol/L and 6.1mmol/L (Jones SC, et al. 1999).

In the critical care scenario, where glucose control is considered important, even in people without diabetes, variability is associated with mortality. In 7049 critically ill subjects the SD of blood glucose concentrations was a significant independent predictor of mortality in the intensive care unit and in hospital (Egi M, et al. 2006). These data have been confirmed by other authors in 3250 subjects with a five-fold mortality increase between the lowest and highest quartiles of standard deviation (Calles-Escandon J, et al. 2010) and in 5728 patients in a study which demonstrated that high variability accompanied by a high mean glucose conferred the highest mortality (Hermanides J, et al. 2010). These data have also been shown in a paediatric intensive care unit where a retrospective review of 1094 patients showed that those in the highest quintile of glycaemic variability had a longer length of stay and significantly elevated mortality (Wintergerst KA, et al. 2006).

In people with stroke, GV has been investigated in people with and without diabetes using finger-prick glucose testing. Increased GV on day 1 after acute ischaemic stroke has been associated with poor functional outcome on hospital discharge but this effect was lost at 3 months follow-up (Camara-Lemarroy et al. 2016.). Early neurological deterioration in acute ischaemic stroke has also been associated with GV (Hui et al. 2018) In people without diabetes, more pronounced stress hyperglycaemic responses measured by continuous glucose monitoring over the initial 72 hours after acute stroke were associated with death or dependency at 3 months (Wada et al. 2018).

## 1.2 Rationale for current study

Increased glycaemic variability is associated with worse outcome in patients with diabetes after acute stroke.

## 1.3 Future Work

We subsequently plan to undertake a prospective randomised control trial utilising real-time continuous glucose monitoring in people with hyperacute stroke and diabetes, to determine if enhanced real-time measurement of glucose levels leads to reduced GV and, hopefully, better outcomes.

# 2. STUDY OBJECTIVES

**Primary Objective**

Establish the impact of glycaemic variability on outcomes in acute stroke in people with and without diabetes.

**Glycaemic Primary outcome:**

Mean absolute glucose (MAG) change

**Stroke Primary outcomes:**

Modified Rankin Scale (MRS) at 90 days

National Institutes of Health Stroke Scale (NIHSS) at 24, 48 and 72 hours

**Glycaemic Secondary outcomes:**

Number hypoglycaemic excursions,

Time (minutes and %) spent in hypoglycaemia (<3.9mmol/L, 70mg/dL)

Time spent in hypoglycaemia (<2.8mmol/L, 50mg/dL)

Time spent in hypoglycaemia (<3.3mmol/L, 60mg/dL)

Time in euglycaemia (3.9-7.8mmol/L, 70-140mg/dL)

Time spent in target (3.9-10mmol/L, 70-180mg/dL)

Time spent in hyperglycaemia (>10mmol/L, 180mg/dL)

Severe hypoglycaemia (3rd party assistance required)

MAD%,

MARD%

Glucose variability, measured by Mean absolute glucose (MAG) and Low blood glucose index (LBGI)

HbA1c and other markers of metabolism

**Stroke Secondary outcomes:**

Barthel Index (BI)

Stroke Impact Scale (SIS)

Pneumonia or infection requiring antibiotic treatment

All cause vascular events (MI, stroke recurrence etc) during first 14 days

Requirement for enteral feeding and duration of enteral feeding

**Other Secondary outcomes:**

Mortality at 90 days

Length of hospital stay

Readmission to hospital

Renal complications

Escalation to High Dependency Unit or Intensive Care Unit

## 3. STUDY DESIGN

This will be an observational cohort study.

100 consecutive volunteers with an acute stroke (NIHSS score >6) and a previous diagnosis of diabetes who are admitted to Charing Cross Hospital Hospital and 100 people with an acute stroke (NIHSS score >6) without diabetes will be recruited in a 1:1 ratio.

Each participant will remain in the study for the duration of their stay in hospital, up to a maximum of 3 months. 1 year total duration for active recruitment and study conduct.

# 4. Participant Entry

## 4.1 Pre-registration evaluations

NIHSS Score (this should be greater than 6). Participants will have been admitted to hospital for an acute stroke.

## 4.2 Inclusion Criteria

- Known Diabetes **or** HbA1c >42 **or** no known diabetes
- Clinically suspected ischaemic stroke (NIHSS score >6) within 12 hours of symptoms
- Adults ≥18 years of age

## 4.3 Exclusion Criteria

- Haemorrhagic stroke
- Unable to participate due to other factors, as assessed by the Chief Investigators
- Pregnancy
- Premorbid MRS >2
- Adult over 80 years of age
- Known to have a terminal condition or conditions that suggest a life expectancy less than 1 year

## 4.4 Withdrawal criteria

- The subject has a serious event related to study
- Investigator initiated discontinuation of study due to participation or equipment concerns
- Withdrawal of consent/assent

Withdrawal will be immediate. Identifiable data already collected with consent will be retained and used in the study. No further data would be collected or any other research procedures carried out on or in relation to the participant.

If a participant is unable to provide verbal consent, assent will be sought from their carer, In line with the Mental Capacity Act 2005 (MCA) which covers research involving adults (aged 16 years and over) who lack capacity in England and Wales. If the participant regains capacity consent will be sought. In this instance, if the participant does not consent to be on the study they will be withdrawn and all data will be destroyed.

Participants who lack capacity at the time of their enrollment into the study will further be withdrawn if:

- they indicate in any way that they want to be withdrawn from the project (for example, if they become upset or distressed)
- any of the Act’s requirements are no longer met.

# 5. adverse events

## 5.1 Definitions

**Adverse Event (AE):** any untoward medical occurrence in a patient or clinical study subject.

**Serious Adverse Event** **(SAE):** any untoward and unexpected medical occurrence or effect that:

- **Results in death**
- **Is life-threatening** – *refers to an event in which the subject was at risk of death at the time of the event; it does not refer to an event which hypothetically might have caused death if it were more severe*
- **Requires hospitalisation, or prolongation of existing inpatients’ hospitalisation**
- **Results in persistent or significant disability or incapacity**
- **Is a congenital anomaly or birth defect**

Medical judgement should be exercised in deciding whether an AE is serious in other situations. Important AEs that are not immediately life-threatening or do not result in death or hospitalisation but may jeopardise the subject or may require intervention to prevent one of the other outcomes listed in the definition above, should also be considered serious.

## 5.2 Reporting Procedures

All adverse events should be reported. Depending on the nature of the event the reporting procedures below should be followed. Any questions concerning adverse event reporting should be directed to the Chief Investigator in the first instance.

**5.2.1 Non serious AEs**

All such events, whether expected or not, should be recorded.

**5.2.2 Serious AEs**

An SAE form should be completed and faxed to the Chief Investigator within 24 hours. However, relapse and death due to stroke (or diabetes in participants with diabetes), and hospitalisations for elective treatment of a pre-existing condition do not need reporting as SAEs.

All SAEs should be reported to the <name of REC> where in the opinion of the Chief Investigator, the event was:

- ‘related’, ie resulted from the administration of any of the research procedures; and
- ‘unexpected’, ie an event that is not listed in the protocol as an expected occurrence

Reports of related and unexpected SAEs should be submitted within 15 days of the Chief Investigator becoming aware of the event, using the NRES SAE form for non-IMP studies. The Chief Investigator must also notify the Sponsor of all SAEs.

Local investigators should report any SAEs as required by their Local Research Ethics Committee, Sponsor and/or Research & Development Office.

**Contact details for reporting SAEs**

[**jrco@imperial.ac.uk**](mailto:jrco@imperial.ac.uk)

**CI email (and contact details below)**

**Fax: 020 3311 1080, attention Dr Neil Hill**

**Please send SAE forms to:**

**Dept Endocrinology and Diabetes,**

**9^th^ Floor, East Block,**

**Charing Cross Hospital,**

**Fulham Palace Road, LONDON W6 8RF**

**Tel: 020 3311 1064 (Mon to Fri 09.00 – 17.00)**

# 6. ASSESSMENT AND FOLLOW-UP

## 6.1 Recruitment and consent

Recruitment will be undertaken in collaboration with the Stroke team of Charing Cross Hospital in London. Participants will be identified via the emergency departments following an acute stroke and participant information sheets will be given to potential subjects. If a participant is unable to provide verbal consent, assent will be sought from their carer, In line with the Mental Capacity Act 2005 (MCA) which covers research involving adults (aged 16 years and over) who lack capacity in England and Wales.

Where possible, Informed consent will be obtained. Hospital research staff will approach the patient to discuss the study and provide a patient information sheet. After allowing sufficient time for a decision about whether to take part in the study and an opportunity to ask questions, consent will be obtained in writing.

When a patient has mental capacity but is unable to sign the consent form (e.g. because of weakness of the dominant hand following stroke), consent is confirmed orally in the presence of a witness (an individual not otherwise involved in the trial), and the witness signs and dates the consent form on behalf of the participant.

It is anticipated that approximately one third of study-eligible patients will be unable to engage with an informed consent process due to the effects of stroke upon communication and cognition. As exclusion of this group would drastically reduce the clinical relevance of the study, if a patient has been identified as eligible but lacks the capacity to consent, a personal or nominated consultee is approached.

Hospital research staff first attempt to identify an appropriate personal consultee (usually the next of kin) in order to discuss the study and provide a consultee information sheet. If a personal consultee is identified, after allowing sufficient time for him/her to consider the patient’s wishes and feelings and an opportunity to ask questions, the consultee is asked to complete a consultee declaration form if he/she believes the patient would have no objection to taking part in the study.

If an appropriate personal consultee cannot be located, an independent clinician (nominated consultee) is asked to confirm that the patient lacks capacity for con- sent, and that study participation would not introduce a risk of harm or be against the patient’s wishes from what is known about the patient’s character and beliefs. The independent clinician signs an independent clinician declaration form concerning study participation.

If the communication or cognitive difficulties that impeded a patient’s ability to provide consent are still present at 90 days after stroke, where a personal consultee provided permission to enter the study, this person will be contacted to complete the 90 day study-specific outcome questions on behalf of the patient. In cases where an independent clinician provided permission for study participation, the participant will be contacted and if unable to respond, a personal consultee will be contacted.

The early mortality rate following acute stroke is approximately 10%. These patients are usually identified soon after admission and treated palliatively. However, unexpected deaths also occur.

When a patient has died, or if a formal palliative end of life care process has been started at the point when the patient is identified as eligible for the study, individual patient consent will not be possible, and it is likely to be distressing for a personal consultee to be approached regarding the research use of routinely collected health- care data. Acute stroke has many effects upon neurological function and consciousness which can fluctuate for several days or even weeks.

However, these are likely to be challenging clinical situations where seeking an alternative method of consent will be difficult because of the severe degree of remaining neurological impairment and the time elapsed since admission. In these unusual scenarios, an alternative method of consent will not be pursued, and the patient will not be included.

## 6.2 Enrollment and study conduct

Following informed consent/assent the research team will collect full medical and medication history, as well as historic bloods from Cerner (hospital computer system) as per routine clinical care. In addition to the admission blood tests, venous blood (<20 ml) will be taken on admission and at 24 and 72 hours for measurement of biochemical, metabolic and hormonal profiles (including, but not limited to: glucose, HbA1c, c-peptide, CRP and other markers of neuro-inflammation). Blood samples (whole blood and plasma) will be stored temporarily in a -20 degree freezer (frozen within 24 hours) at Charing Cross Hospital and transported in bulk by courier on dry ice to a -80 freezer at St Mary Hospital for the use in future research. If participants meet the inclusion criteria they will be enrolled on to the study immediately.#

We do not anticipate that any significant incidental findings will arise as the data pulled from Cerner will be retrospective and much of the sample analysis is done post-hoc. If participants in the “non-diabetes” cohort return blood results that indicate that they have diabetes we will inform their clinical team so that they can manage this appropriately.

Blinded continuous glucose monitoring (CGM) will be applied to 100 consecutive volunteers with an acute stroke (NIHSS score >6) and a previous diagnosis of diabetes who are admitted to Charing Cross Hospital and 100 people with an acute stroke (NIHSS score >6) without diabetes in a 1:1 ratio. This will be done as soon as is feasible after admission. Clinical team will be instructed to test participants capillary blood glucose as per standard care, and if symptoms of hypo- or hyperglycaemia occur. Sensors will be worn for a maximum of 10 days (whilst in hospital) but will be removed on hospital discharge should this occur before 10 days are up. Glucose alerts will not be enabled as the CGM will be blinded.

CGM involves insertion of a small plastic cannula to the subcutaneous tissue of the abdominal skin by members of the study team. The cannula is attached to a small data logger which will be taped to the patients’ skin. CGM will take place for 10 days, throughout the participants stay in hospital.

If the participants’ sensor fails or falls out the research team will provide a new sensor and will help the participant to insert it. .

Recent data has demonstrated that the Dexcom G6 sensor and data logger are able to go through an MRI machine so if the participant requires an MRI scan as part of their clinical care (independent to the study) they can wear the sensor and data logger throughout.

## 6.3 Telephone follow up at 3 months

Participants who are discharged from the hospital within 3 months will be followed up by the research team via a telephone call (in order to obtain MRS scores).

Participants will be given the option to provide consent to be contacted again for future research.

End of study will be defined as Last Subject Last Visit (LSLV)

7. STATISTICs and data analysis

This is a hypothesis generating study and, as such, we are not proposing a fully-powered study. The data gathered from the proposed work will serve as robust pilot data to support a larger randomised study. At the end of the study, participants with impaired glucose tolerance or diabetes will be sub analysed.

Parametric data will be compared using an independent t-test. Non-parametric data between groups will be compared using a Mann-Whitney test.

Data and all appropriate documentation will be stored for a minimum of 10 years after the completion of the study, including the follow-up period.

# 8. regulatory issues

## 8.1 Ethics approval

The Study Coordination Centre has obtained approval from the xxx Research Ethics Committee (REC) and Health Regulator Authority (HRA). The study must also receive confirmation of capacity and capability from each participating NHS Trust before accepting participants into the study or any research activity is carried out. The study will be conducted in accordance with the recommendations for physicians involved in research on human subjects adopted by the 18th World Medical Assembly, Helsinki 1964 and later revisions.

## 8.2 Consent

Consent to enter the study must be sought from each participant only after a full explanation has been given, an information leaflet offered and time allowed for consideration. Signed participant consent should be obtained. The right of the participant to refuse to participate without giving reasons must be respected. After the participant has entered the study the clinician remains free to give alternative treatment to that specified in the protocol at any stage if he/she feels it is in the participant’s best interest, but the reasons for doing so should be recorded. In these cases the participants remain within the study for the purposes of follow-up and data analysis. All participants are free to withdraw at any time from the protocol treatment without giving reasons and without prejudicing further treatment. Refer to section on recruitment and consent for further details.

## 8.3 Confidentiality

The Chief Investigator will preserve the confidentiality of participants taking part in the study and is registered under the Data Protection Act. Confidentiality of data will be preserved during data transmission via the use of anonymised codes. Participants will be assigned codes based on order of enrolment and site at which they are enrolled (G-VAS-001 etc.). Identifiable personal information will be stored securely in a locked room at Imperial College Healthcare NHS Trust and Imperial College London. Pseudonymised data will be stored on Imperial College Healthcare NHS Trust computers, Imperial college London computers or encrypted laptop computers (either ICL/ICHT). Data will be accessible only to the Research Team and for audit. Pseudonymised data will be used for analysis. The data will be stored at Imperial College London for 10 years. The data custodian is Dr Neil Hill.

## 8.4 Indemnity

Imperial College London holds negligent harm and non-negligent harm insurance policies which apply to this study

## 8.5 Sponsor

Imperial College London will act as the main Sponsor for this study. Delegated responsibilities will be assigned to the NHS trusts taking part in this study.

## 8.6 Funding

Dexcom are funding this study. No staff or participants will receive any reimbursements for this study.

## 8.7 Audits

The study may be subject to inspection and audit by Imperial College London under their remit as sponsor and other regulatory bodies to ensure adherence to GCP and the UK Policy Frame Work for Health and Social Care Research.

# 9. Study Management

The day-to-day management of the study will be co-ordinated through Dr Neil Hill.

**10. PUBLICATION POLICY**

The study will be registered on the clinicaltrials.gov system and results will be disseminated by peer reviewed scientific journals, internal report, conference presentation and publication on websites. No identifiable personal data will be published. All anthropometry and personal clinical data will be expressed as mean/ median and spread of the population in the study. Details of any publications that arise from the study will be disseminated to participants.

# 11. References

American Heart Association. Stroke Hyperglycaemia Insulin Network Effort (SHINE) Trial. 2019. Available from: https://professional.heart.org/professional/ScienceNews/UCM_503415_SHINE-Clinical-Trial-Details.jsp

Bellolio MF, et al. Insulin for glycaemic control in acute ischaemic stroke. Cochrane Database Syst Rev 2014;(1):CD005346

Burton JK, Terence J Quinn TJ, Miles Fisher M. Diabetes and stroke. Practical Diabetes. AUGUST 1, 2019 [VOL 36.4 JULY/AUGUST 2019](https://www.practicaldiabetes.com/year-volume-issues/vol-36-4-july-august-2019/)

Calles-Escandon J, Lovato LC, Simons-Morton DG, Kendall DM, Pop-Busui R, Cohen RM, et al. Effect of intensive compared with standard glycemia treatment strategies on mortality by baseline subgroup characteristics: the Action to Control Cardiovascular Risk in Diabetes (ACCORD) trial. Diabetes Care. 2010;33:721–727

Camara-Lemarroy C, González-Moreno E, Garza-Villarreal E, Treviño-Herrera A, Tena-Montiel R, Muruet W, Gongora Rivera J. Glycemic Variability and Functional Outcome After Acute Ischemic Stroke. American Academy of Neurology. 2016;86:P1.191

Egi M, Bellomo R, Stachowski E, French CJ, Hart G. Variability of blood glucose concentration and short-term mortality in critically ill patients. Anesthesiology. 2006;105:244-252

Hermanides J, Vriesendorp TM, Bosman RJ, Zandstra DF, Hoekstra JB, Devries JH.

Glucose variability is associated with intensive care unit mortality. Crit Care Med. 2010;38:838-842

Hui J, Zhang J, Mao X, Li Z, Li X, Wang F, Wang T, Yuan Q, Wang S, Pu M, Xi G. The initial glycemic variability is associated with early neurological deterioration in diabetic patients with acute ischemic stroke. Neurol Sci. 2018;39:1571-1577

Jones SC, Saunders HJ, Qi W, Pollock CA. Intermittent high glucose enhances cell growth and collagen synthesis in cultured human tubulointerstitial cells. Diabetologia. 1999;42:1113-1119

Masrur S, et al. Association of acute and chronic hyperglycemia with acute ischemic stroke outcomes post-thrombolysis: Findings from Get With The Guidelines-Stroke. J Am Heart Assoc 2015;4:e002193

Muir KW, et al. Prevalence, predictors and prognosis of post-stroke hyperglycaemia in acute stroke trials: Individual patient data pooled analysis from the Virtual International Stroke Trials Archive (VISTA). Cerebrovasc Dis Extra. 2011;1:17–27

National Institute for Health and Care Excellence. CG68: Stroke and transient ischaemic attack in over 16s: diagnosis and initial management. 2017. Available from: https://www.nice.org.uk/guidance/cg68

Piconi, L., Quagliaro L, Da Ros R, Assaloni R, Giugliano D, Esposito K, Szabó C, Ceriello A. Intermittent high glucose enhances ICAM‐1, VCAM‐1, E‐selectin and interleukin‐6 expression in human umbilical endothelial cells in culture: the role of poly(ADP‐ribose) polymerase. Journal of Thrombosis and Haemostasis. 2004;2:1453-1459

Quagliaro L, Piconi L, Assaloni R, Martinelli L, Motz E, Ceriello A. Intermittent high glucose enhances apoptosis related to oxidative stress in human umbilical vein endothelial cells: the role of protein kinase C and NAD(P)H-oxidase activation. Diabetes. 2003;52:2795-804

Risso A, Mercuri F, Quagliaro L, Damante G, Ceriello A. Intermittent high glucose enhances apoptosis in human umbilical vein endothelial cells in culture. Am J Physiol Endocrinol Metab. 2001;281:E924-30

Wada S, Yoshimura S, Inoue M, Matsuki T, Arihiro S, Koga M, Kitazono T, Makino H, Hosoda K, Ihara M, Toyoda K.Outcome Prediction in Acute Stroke Patients by Continuous Glucose Monitoring. J Am Heart Assoc. 2018:12;7

Wintergerst KA, Buckingham B, Gandrud L, Wong BJ, Kache S, Wilson DM. Association of hypoglycemia, hyperglycemia, and glucose variability with morbidity and death in the pediatric intensive care unit. Pediatrics. 2006;118:173-179

# 12. APPENDICES

## Appendix 1. Summary of investigations, treatment and assessments


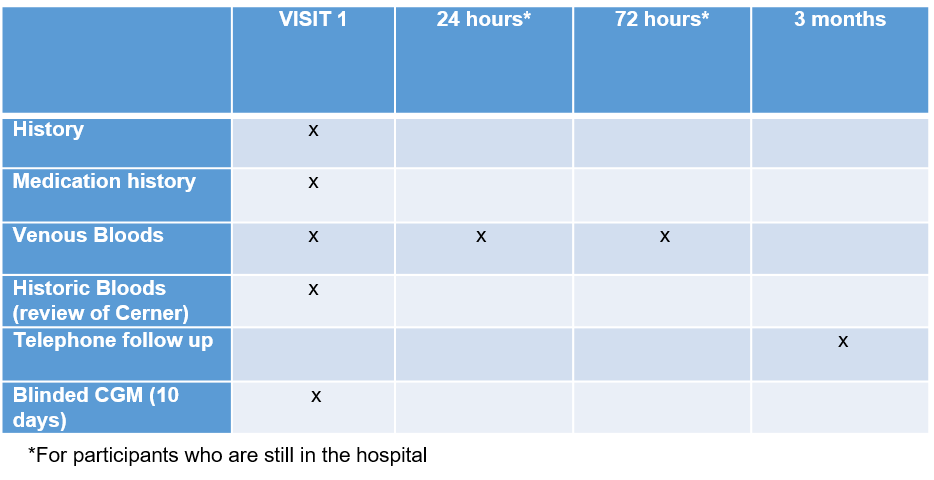


## Appendix 2. Data Flow Mapping


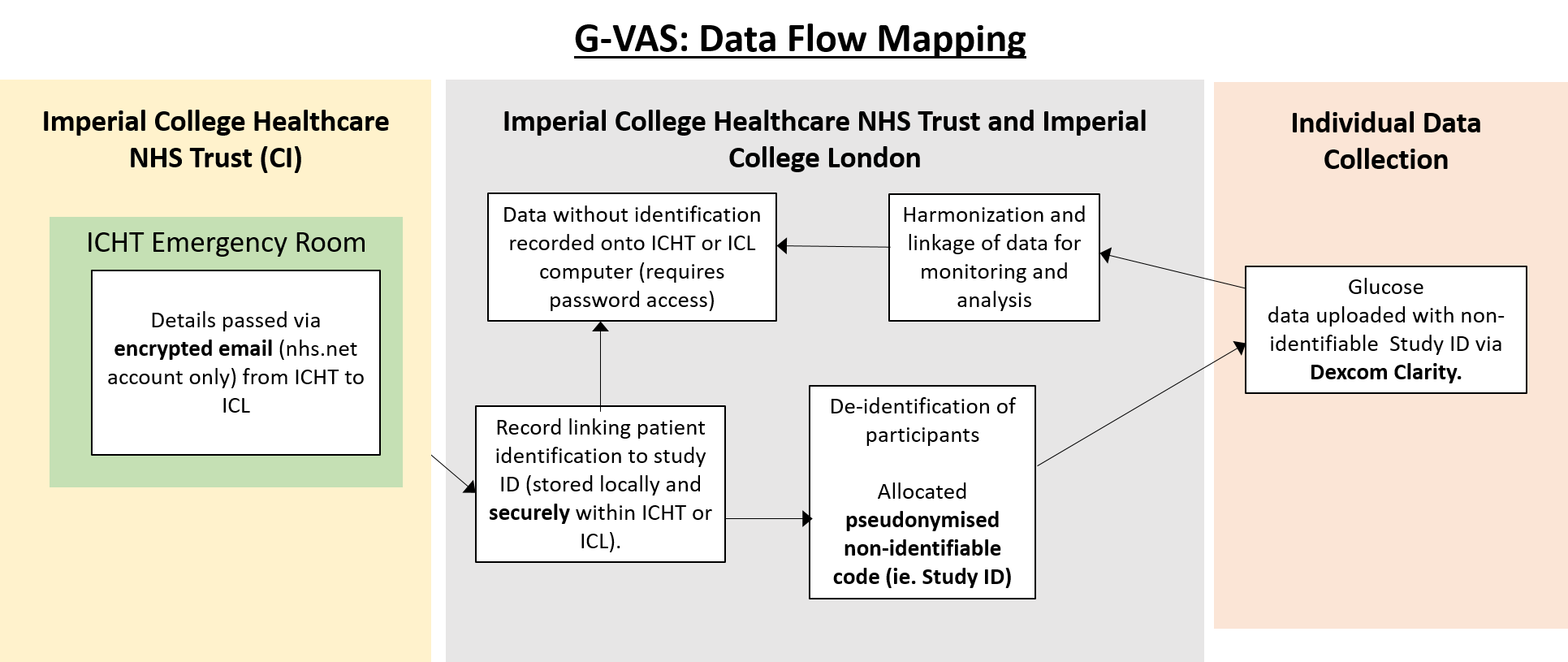

Supplement: S1 File — (DOCX) [file pone.0318456.s001.docx]
