# Supplementary material for: Glycaemic level and glycaemic variability in acute ischaemic stroke and functional outcome: An observational continuous glucose monitoring study
Source: PLoS One. 2025 May 30;20(5):e0318456. doi: 10.1371/journal.pone.0318456 (PMC12124563; doi:10.1371/journal.pone.0318456)
Supplement: Attachment — Submitted filename: 241024 GVAS Response to Reviewers.docx [file pone.0318456.s003.docx]

**Reviewer #1:** This study investigates the relationship between glycaemic variability and functional outcomes in acute ischaemic stroke patients, finding that stroke severity at admission, rather than glycaemic variability, is the key predictor of functional deterioration at discharge.

While the study offers valuable clinical insights into the relationship between glycaemic variability and functional outcomes in acute ischaemic stroke patients, several limitations should be addressed.

1. The authors initiated glucose monitoring at an average of 38.4 hours after stroke onset, which may have missed short-term glycaemic fluctuations immediately following acute stroke.

Yes, this is a limitation that we struggled to overcome. We had anticipated that we would be able to recruit patients in the Emergency Department and initiate CGM immediately but the reality was, due to a number of factors including but not limited to; challenges seeking consent/assent in the acute setting, clinical expedience, other studies, availability of study personnel to recruit, weekend and overnight admissions, timing of MRI scans, all combined to mean that we failed to start the CGM as promptly as hoped.

2. The sample size of this study needs to be bigger, with only 67 valid cases, which is insufficient to draw convincing conclusions.

Agree – as mentioned above, we struggled with recruitment and were further hampered by the Covid-19 pandemic (page 7, lines 129-131). Whilst 67 is less than we had hoped for it remains a reasonably sized cohort and I hope we have been sufficiently cautious in our interpretation of the data given the number of participants. I’ve reiterated this further in the discussion and highlighted this with the following sentence:

‘Challenges with recruitment and a smaller than intended cohort means that the inferences gained from the data should be treated with due caution. Although this was intended as a hypothesis generating study, a greater number of participants would have made our findings more robust.’

3. Diabetes is a risk factor for stroke, and combining glycaemic metrics from stroke patients with diabetes and those without diabetes in the same analysis may be inappropriate.

We did an analysis of only the people without diabetes, comparing those who remained independent with those who deteriorated to dependent (ie the same analysis as included in Table 2) which I have added below (‘No diabetes’), with the whole cohort’s data (‘Total’) included for comparison. The CGM metrics are similar in the Total group and No diabetes groups; we felt that including the Total group incorporated the richer data. I have included a line in the Results as follows:

‘Comparison of the glucose metrics in the cohort without diabetes (n=42) who either remained independent or deteriorated to dependent showed similar patterns to that of the wider group (with and without diabetes, n=54).’

However, if you would like me to include this table as Supplementary Data we would be happy to do that too.

|  | **Total** | | | **No diabetes** | | |
| --- | --- | --- | --- | --- | --- | --- |
|  | **Remained Independent**  **(n=17)** | **Deteriorated to dependent**  **(n=37)** | ***p*-value** | **Remained Independent**  **(n=16)** | **Deteriorated to dependent**  **(n=26)** | ***p-*value** |
| Age (years) | 62.6±13.5 | 74.4±12.5 | 0.003 | 62.5±14 | 73.6±13.4 | 0.02 |
| Male gender, n (%) | 11 (64.7) | 25 (67.6) | 0.83 | 10 (62.5) | 18 (69.2) | 0.65 |
| Body mass index (kg/m^2^)^b^ | 27.6±5.9 | 26.6±3.9 | 0.54 | 27.6±6 | 26.2±4 | 0.46 |
| SBP at arrival (mmHg) | 146±15 | 160±30 | 0.03 | 145±15 | 154±28 | 0.17 |
| DBP at arrival (mmHg) | 88±14 | 90±15 | 0.72 | 88±14 | 90±15 | 0.65 |
| Smoking, n (%) | 6 (35.3) | 9 (24.3) | 0.52 | 6 (37.5) | 7 (26.9) | 0.51 |
| MRS pre-admission | 0 (0,1) | 1 (0,1) | 1.00 | 0 (0,1) | 0 (0,1) | 0.75 |
| NIHSS at admission | 5 (2,8) | 9 (4,16.5) | 0.01 | 4 (2,8) | 10.5 (5,17) | 0.004 |
| Reperfusion therapy, n (%) | 6 (35.3) | 15 (40.5) | 0.71 | 6 (37.5) | 10 (38.5) | 0.95 |
| NIHSS 24 hours after intervention (n=23) | 1.5 (1,4) | 9 (3,15) | 0.01 | 1.5 (1,4) | 6.5 (3,13) | 0.03 |
| Glucose lowering agents^c^, n (%) | 1 (5.9) | 8 (21.6) | 0.24 | 1 (6.3) | 1 (3.8) | 1.00 |
| Enteral tube feeding^c,^ n (%) | 0 | 4 (10.8) | 0.30 | 0 | 4 (15.4) | 0.28 |
| Antibiotic treatment^c^, n (%) | 1 (5.9) | 12 (32.4) | 0.04 | 0 | 8 (30.8) | 0.02 |
| Comorbidities | | | | | | |
| -Diabetes, n (%) | 1 (5.9) | 11 (29.7) | 0.08 | - | - | - |
| -Hypertension, n (%) | 8 (47.1) | 23 (62.2) | 0.30 | 7 (43.8) | 12 (46.2) | 0.88 |
| -Congestive heart failure, n (%) | - | 6 (16.2) | 0.16 | - | 3 (11.5) | 0.28 |
| -Atrial fibrillation, n (%) | 1 (5.9) | 14 (37.8) | 0.02 | 1 (6.3) | 8 (30.8) | 0.12 |
| -Atherosclerosis | 7 (41.2) | 14 (37.8) | 0.82 | 6 (37.5) | 9 (34.6) | 0.85 |
| Glycemic parameters | | | | | | |
| - Blood glucose at admission, (mg/dL) | 110 (99,140) | 121 (92,166) | 0.75 | 108 (98,136) | 103 (90,132) | 0.56 |
| - HbA1c at admission (%) | 5.8 (5.4,6.2) | 6.0 (5.5,6.8) | 0.40 | 5.6 (5.3,6.0) | 5.6 (5.4,6.0) | 0.80 |
| CGM data during first 72 hours after admission | | | | | | |
| Mean glucose (mg/dL) | 121 (97,135) | 133 (121,158) | 0.02 | 119 (37,133) | 125 (119,145) | 0.06 |
| SD | 1.02 (0.85,1.43) | 1.49 (1.00,1.94) | 0.04 | 1.00 (0.82,1.31) | 1.31 (0.95,1.65) | 0.04 |
| % CV | 17.0 (12.7,20.3) | 18.3 (13.2,23.5) | 0.33 | 16.9 (12.2,20.3) | 18.4 (14.2,23.4) | 0.33 |
| CONGA | 1.51 (1.17,1.87) | 1.76 (1.29,2.23) | 0.13 | 1.51 (1.14,1.73) | 1.63 (1.16,2.26) | 0.26 |
| LBGI | 0.25 (0.04,2.12) | 0.28 (0.00,0.35) | 0.11 | 0.53 (0.04,2.12) | 0.13 (0.03,0.42) | 0.24 |
| MAGE | 2.85 (2.17,3.48) | 4.31 (2.42,4.87) | 0.07 | 2.76 (2.15,3.45) | 3.70 (2.28,4.59) | 0.12 |
| MAG | 2.61 (2.14,3.74) | 2.47 (2.04, 3.05) | 0.41 | 2.76 (2.10,3.91) | 2.41 (1.94,2.97) | 0.20 |
| % TIR 70-180 mg/dL | 96.7 (91.0,99.7) | 87.8 (75.6,97.5) | 0.12 | 97.0 (93.2,99.8) | 93.9 (83.8,99.7) | 0.24 |
| % TBR < 70 mg/dL | 0 (0.0,3.0) | 0 (0.0,0.7) | 0.29 | 0 (0.0,3.1) | 0 (0.0,1.2) | 0.32 |
| % TBR < 54 mg/dL | 0 (0.0,1.4) | 0 | 0.39 | 0 (0,1.5) | 0 | 0.29 |
| % TAR > 180 mg/dL | 0 (0.0,3.9) | 8.0 (0,15.5) | 0.03 | 0 (0,1.9) | 4.6 (0,12.3) | 0.09 |
| Length of stay in stroke unit, days | 3.1 (2.0,4.7) | 3.4 (2.6,6.2) | 0.19 | 3.0 (2.0,4.6) | 3.4 (2.4,7.1) | 0.15 |
| Re-admission, n (%) | 2 (12.5) | 4 (11.8) | 1.00 | 2 (13.3) | 1 (4.0) | 0.55 |
| Death, n (%) | 0 | 4 (10.8) | 0.30 | 0 | 1 (3.8) | 1.00 |

4. The article does not categorize the types of ischaemic stroke, even though different types may respond differently to hyperglycaemia and glycaemic variability. Thank you for raising this. We did classify the types of stroke into cardio-embolic, large artery atherosclerosis, small vessel occlusion and stroke of unknown cause (Table 1) using the Trial of ORG 10172 in Acute Stroke Treatment (TOAST) classification chose not to analyse these groups separately due to the small numbers (and we had not, a priori, planned to).

5. The glucose monitoring methods may need to be more consistent, as factors such as pre- and post-prandial glucose levels or other interventions like intravenous fluids can significantly affect glucose metrics. Additionally, the use of various medications after stroke, many of which can impact blood glucose levels, was excluded from consideration by the authors.

We documented enteral feeding, infection, glucose lowering agents in the different groups (Table 2). Only infection had significant higher number in DD group, which was adjusted in multivariate analysis. Interestingly the (non-significantly) greater use of antibiotics, additional glucose lowering agents and enteral feeding in the DD and RD groups did not correspond with longer duration of time on HASU.

**Reviewer #2:** The authors performed a study evaluation the association between glycemic variability and functional outcome. I have the following comments:

1. Methods – please elaborate more on Enter method. Did the authors use the forward or backward or both directions to obtain results from the multivariate analysis?

We used the forward method. Only significant data from the univariate analysis was carried forwards for the multivariate analysis.

2. Table 2 – why P-value only for RI vs DD? Sorry, I don’t think we clarified this well in the text. The aim of this analysis was to investigate factors that may have contributed or been associated with functional deterioration (or lack of) hence the statistical analysis was undertaken in patients who were, pre-stroke, independent. The data for people who were dependent prior to their admission on this occasion were included for interest and comparison. I have added a sentence clarifying this:

‘Compared to participants in the RI group, those in the DD group were older, with higher SBP, % atrial fibrillation, stroke severity at admission and after reperfusion therapy, and infection rate’ and ‘Clinical characteristics and glycaemic parameters between groups of functional status at discharge are shown in Table 2 (the data for participants who were functionally dependent prior to admission are also included for information).’

3. Did the authors account for other inpatient medical comorbidities? E.g. infection / aspiration pneumonia, a known associated comorbidity after stroke, and their subsequent effect of stress-induced hyperglycemia? E.g. When patient was kept NPO, and thus may have contributed to lower levels of glucose? E.g. when patient had poor oral intake after stroke and was dehydrated so solutions containing glucose e.g. 5% dextrose solution was used to rehydrate the patient?

We documented enteral feeding, infection, and glucose lowering agents in the different groups (Table 2). Unfortunately, we did not collect information on the duration or number of times when patients were kept nil-by-mouth and this is a limitation of our work. To address this I have added the following sentences in the discussion:

‘A further limitation is that we did not record periods when patients were nil-by-mouth; subsequent use of glucose-containing intravenous fluids may have affected their GV’ and ‘It was not possible to determine the effects of infection and associated stress hyperglycaemia but it is possible that this contributed to the increased GV in the DD group’.

**Reviewer #3:** "Glycaemic level and glycaemic variability in acute ischaemic stroke and functional outcome at discharge: an observational continuous glucose monitoring study"
General Overview:
This study aims to explore the relationship between glycaemic variability (GV) and functional outcomes in patients with acute ischaemic stroke (AIS), utilizing continuous glucose monitoring (CGM). The study finds that while GV metrics such as mean glucose and glucose standard deviation (SD) were higher in patients with functional deterioration, only the National Institutes of Health Stroke Scale (NIHSS) at admission was independently associated with worsening functional status in multivariate analysis.
Strengths:
1. Timely Topic: The exploration of GV in the context of stroke is highly relevant, as it contributes to an ongoing discussion about the role of glycaemia in stroke outcomes, especially in light of past conflicting findings on glucose management.
2. Use of Continuous Glucose Monitoring (CGM): The use of CGM provides more granular glucose data than traditional point-of-care tests, adding a novel aspect to the research.
3. Statistical Rigor: The manuscript employs a variety of appropriate statistical techniques, such as logistic regression, and controls for multiple variables in the multivariate analysis.
4. Ethical Approvals and Patient Consent: The study has clear ethical protocols and consent processes, which are meticulously documented.

Major Issues:
1. Study Design and Timing of CGM Initiation:
o Issue: The CGM was initiated at 38.4 hours after stroke onset, potentially missing critical early glucose fluctuations. The study acknowledges this limitation but does not adequately address how it affects the interpretation of GV’s role in the early phases of stroke.
o Recommendation: A deeper discussion about the impact of this delay in CGM initiation on glucose variability (especially hyperglycaemia in the acute phase) would strengthen the manuscript. The authors could explore sensitivity analyses or reference studies where earlier glucose monitoring was performed.

Thank you for your comments and suggestions – this has been very helpful for us in attempting to address the issues you have raised.

We have added the following (in red, below the pre-existing text in black) about the impact of delayed CGM initiation, and added further discussion in response to your suggestion to explore the literature more thoroughly in Minor Issues point 2 below.

‘Therefore, it is possible that early-phase hyperglycaemia and GV more immediately post stroke was missed. A previous CGM study suggested that there was 29 an early-phase of hyperglycaemia during 8 hours after stroke onset, followed by a decrease in glucose at 14-16 hours poststroke, and finally a late-phase hyperglycaemia at 48-88 hours post stroke. Thus, missing early-phase glucose fluctuation in our study might have attenuated the observed relationships between GV and stroke outcomes, and potentially resulting in non-significant results in the multivariate logistic regression analysis.’

We also looked at our data again, by tertiles (table below), to see whether the glucose data were affected by start-time of CGM. Only conga was noted to be significantly different amongst the GV metrics.

|  | **1^st^ tertile**  **(0-<31 hours)**  **(n=22)** | **2^nd^ tertile**  **(31-<44 hours)**  **(n=23)** | **3^rd^ tertile**  **(≥ 44 hours)**  **(n=22)** | **P value** |
| --- | --- | --- | --- | --- |
| Mean glucose (mg/dL) | 128 (117,145) | 125 (113,148) | 146 (124,187) | 0.09 |
| SD | 1.25 (0.94, 1.57) | 1.32 (0.90, 1.82) | 1.21 (1.00, 2.35) | 0.47 |
| % CV | 18.5 (13.7, 21.3) | 18.6 (13.2, 22.3) | 16.2 (13.5, 23.7) | 0.91 |
| CONGA^a^ | 1.45 (1.05, 1.62) | 1.80 (1.35, 2.44) | 1.82 (1.47, 2.09) | 0.03 |
| LBGI | 0.19 (0.01, 1.20) | 0.16 (0.03, 0.82) | 0.03 (0.00, 0.13) | 0.06 |
| MAGE^b^ | 3.11 (2.04, 3.56) | 4.06 (2.83, 4.53) | 3.93 (2.44, 5.35) | 0.12 |
| MAG | 2.31 (1.87, 2.99) | 2.79 (1.93, 3.15) | 2.56 (2.36, 3.13) | 0.25 |
| % TIR 70-180 mg/dL | 92.4 (85.7, 98.0) | 93.7 (75.6,99.5) | 88.5 (47.7, 99.1) | 0.68 |
| % TBR < 70 mg/dL | 0 (0, 4.9) | 0 (0,1.4) | 0 | 0.06 |
| % TBR < 54 mg/dL | 0 (0,0.9) | 0 | 0 | 0.10 |
| % TAR > 180 mg/dL | 1.6 (0,11.1) | 4.2 (0,15.2) | 8.1 (0.5,51.0) | 0.29 |

a. CONGA was calculated from 63 patients
b. MAGE was calculated from 59 patients

2. Sample Size and Power:
o Issue: The sample size (67 participants) is relatively small, especially given that the study aims to explore associations with multiple variables (NIHSS, glucose variability, comorbidities). Although the authors attribute the limited sample to the impact of COVID-19 on recruitment, this does raise concerns about the study’s power.
o Recommendation: The authors should discuss the implications of this limited sample size more explicitly. A post-hoc power analysis would be beneficial to show whether the sample size was adequate to detect significant associations, particularly for GV metrics like SD and MAGE.

We have enlisted the support of a statistician to review the manuscript and ensure any statistical queries are appropriately addressed. We added further discussion to address the issue of low number of events that may have affected the results of multivariate model and added the following sentence:

‘Even though mean glucose and SD were higher in DD group, multivariate analysis did not find the association of glucose level and GV on functional outcome. This might be explained by the strong association of stroke severity (NIHSS) and functional outcome and low number of events in the multivariate model compared to adjusting factors.’

We have, nevertheless, followed the reviewer's suggestion to undertake post hoc power calculations (see table below), using the data presented in our manuscript as pilot study information, which could provide some perspective on the strength of our conclusions. Treating each outcome variable as a separate primary outcome, to have detected the differences summarised in Table 2 as significant at p<0.05 and with 80% power, we would have needed between 80 and 238 participants (for mean glucose concentration and CONGA, respectively, with equal numbers in each group). A fully powered two-group comparison, would have required for SD 88 and MAGE 100. Powering on the basis of the univariable logistic regression findings (Table 3) generally gave similar numbers (82 for SD and 104 for MAGE). Those are, of course, the minimum numbers needed, minimised by nominating each outcome variable as an alternative primary outcome. If the intention was to evaluate more than one variable in a fully powered study, Bonferroni corrections for multiple testing would be needed, depending on the numbers of outcome variables the study was intended to cover. These estimates make it clear that the numbers we recruited fell somewhat short of the numbers needed for a fully powered study and our study must, therefore, be regarded as essentially a pilot study. We have included these post hoc estimates at the end of our revised Results section:

‘We undertook post hoc power calculations, using the data presented in our manuscript as pilot study information, to provide perspective on the strength of our conclusions. Treating each outcome variable as a separate primary outcome, to have detected the differences summarised in Table 2 as significant at p<0.05 and with 80% power, we would have needed between 80 and 238 participants (for mean glucose concentration and CONGA, respectively, with equal numbers in each group). A fully powered two-group comparison, would have required for 88 participants for SD and 100 participants for MAGE. Powering on the basis of the univariable logistic regression findings (Table 3) gave similar numbers (82 for SD and 104 for MAGE). Those are the minimum numbers needed, minimised by nominating each outcome variable as an alternative primary outcome. If the intention was to evaluate more than one variable in a fully powered study, Bonferroni corrections for multiple testing would be needed, depending on the numbers of outcome variables the study was intended to cover.’

We also noted their implications in our revised Discussion:

‘Our post-hoc power calculations indicate that we would have required 40 people in each group to identify a difference in mean glucose between RI and DD, and 52 people to detect a difference in MAGE, and 44 participants for SD.’

| Variable | Two-Group Comparison | Logistic Regression |
| --- | --- | --- |
| Mean glucose | 80 | 57 |
| SD | 88 | 82 |
| CV | 160 | 125 |
| CONGA | 238 | 281 |
| MAGE | 100 | 104 |
| MAG | 174 | 422 |

3. Generalizability:
o Issue: The study includes a highly specific population, with 13.4% of patients suffering from lacunar infarctions, which the authors acknowledge might dilute the effect of GV. The broader inclusion criteria (no distinction between stroke subtypes) limit the ability to draw conclusions about certain stroke populations, particularly those with large vessel occlusion.
o Recommendation: The authors should stratify results based on stroke subtype or provide an analysis of how subtype heterogeneity impacts the findings. Subgroup analyses may offer more insights into whether GV plays a more significant role in specific types of strokes.

We agree that further studies aiming to explore the association of specific stroke subtype and GV might provide clearer picture of the association. However, because our study had small sample size, we elected not to perform sub analysis according to stroke subtype.

4. Glycaemic Variability Metrics:
o Issue: While multiple GV metrics were calculated (SD, CV, MAGE, MAG, etc.), the manuscript does not sufficiently discuss why certain metrics might be more meaningful than others in the context of stroke outcomes. For instance, SD is discussed more thoroughly than MAGE, even though the latter is a more direct measure of fluctuations.
o Recommendation: The authors should justify their choice of metrics more robustly. A clearer explanation of why SD and MAGE were prioritized, along with a detailed examination of other GV metrics (CV, CONGA), would be helpful.

- Thank you for your important comment. The following has been added to the methods section: These metrics were included to reflect the previous literature describing glucose variability and to include an overall measure of spread (SD), a measure of spread independent of the mean glucose (%CV), a ‘distance-travelled’ metric that takes account of time (MAG), a short-term measure of glucose change (CONGA), and a metric sensitive to hypoglyaemic risk (LBGI). MAG has been previously reported to be associated with adverse outcomes in hospital ([10.1097/CCM.0b013e3181cc4be9](https://doi.org/10.1097/ccm.0b013e3181cc4be9)), and is a good discriminator of variability between individuals (<https://doi.org/10.1089/dia.2019.0415>).

5. Interpretation of Multivariate Analysis:
o Issue: The multivariate analysis finds that NIHSS at admission is the only variable significantly associated with functional deterioration. The failure of GV metrics to show significant associations in this analysis is important, but the authors don’t adequately discuss potential reasons for this.
o Recommendation: The manuscript would benefit from a more thorough discussion of why GV, despite being linked to deterioration in univariate analysis, fails to show significance in the multivariate model. Consideration of potential collinearity between NIHSS and glucose levels or other confounders should be discussed.

Thank you for highlighting this important issue. We explored the collinearity issue of variables used in multivariate model. All variables had Variance Inflation Factor (VIF) below 10 and Collinearity Tolerance above 0.1. We have added the following interpretation in the Discussion:

‘This might be explained by the strong association of stroke severity (NIHSS) and functional outcome and low number of events in the multivariate model compared to adjusting factors.’

Minor Issues:
1. Clarity in Statistical Presentation:
o The statistical methods used are sound, but some results could be presented more clearly. For example, confidence intervals for odds ratios in Table 3 should be consistently formatted and displayed to make interpretation easier.

I have amended the errors in the table
o It would also be helpful to present exact p-values (rather than just stating "<0.05") to provide a better sense of the strength of associations.

Thanks, I’ve changed this too

2. References to Previous Literature:
o While the authors reference many relevant studies, the discussion could engage more critically with past literature, particularly studies that show conflicting results on the role of GV in stroke outcomes. A more detailed comparison with these studies would contextualize the present findings more effectively.

We have added the following to the discussion:

‘Participants in a study by Palaiodimou et al, had similar baseline characteristics to our study including CGM initiation’s time (32 vs 38.4 hours after stroke onset), NIHSS (9 vs 8), and number of patients without diabetes (81 vs 71%). They did not find an association between GV and functional independence or excellent functional outcome at 3 months. However, they identified a significant inverse association of greater mean absolute glucose (MAG) and lower likelihood of neurological improvement during hospitalization before and after adjusting for potential confounders. This study included both acute ischemic and hemorrhagic stroke with younger age of participants (65 vs 72 years) and anatomical location of stoke rather than stroke severity (NIHSS) was used to adjust their multivariate analysis. These factors may explain differing results compared to our study.’

3. Explanation of Study Limitations:
o The limitations section is adequately addressed but could be expanded. For example, the authors should emphasize the challenges posed by the study’s single-center design, which may limit generalizability to other stroke units or hospitals with different practices.

Thank you - I have included this sentence in the discussion:

‘The study recruited from a single centre which may limit wider interpretation of the results to other stroke units or hospitals with different practices.’

4. Data Availability Statement:
o The manuscript states that data availability is restricted due to medical confidentiality, which is acceptable. However, the exact procedure for accessing data should be spelled out more clearly to facilitate reproducibility for other researchers.

The raw anonymised data can be obtained by emailing the corresponding author. I have added this to the text
