# Supplementary material for: Glycaemic level and glycaemic variability in acute ischaemic stroke and functional outcome: An observational continuous glucose monitoring study
Source: PLoS One. 2025 May 30;20(5):e0318456. doi: 10.1371/journal.pone.0318456 (PMC12124563; doi:10.1371/journal.pone.0318456)
Supplement: Attachment — Submitted filename: 250110 GVAS Response to Reviewers.docx [file pone.0318456.s004.docx]

Reviewer #3:

**Title**
The title reflects the study design as observational and mentions the key variables. However, it could be more concise without sacrificing clarity. Consider omitting "at discharge" or merging key elements to improve readability.

Modified as suggested

**Abstract**
Page 2, Lines 22–42: While the abstract is structured well, the conclusions lack nuance. The assertion that glycaemic variability is not associated with functional deterioration seems too definitive for a pilot study with acknowledged limitations. Suggest softening language to reflect the exploratory nature of findings.

Thank you – I’ve done this – changed from ‘Glycaemic variability measured by continuous glucose monitoring started on the second day was not associated with functional deterioration after acute ischaemic stroke’ to ‘In this pilot study, an association between glycaemic variability and functional deterioration after acute ischaemic stroke was not observed’.

The background section could briefly mention why CGM was chosen over traditional glucose monitoring methods for added context.

Added ‘…to ensure all episodes of hyper and hypoglycaemia were captured’.

**Introduction**
Page 4, Lines 55–80: The introduction effectively contextualizes the study but needs clarity in explaining why continuous glucose monitoring is uniquely advantageous.

Added ‘CGM was utilised to ensure a comprehensive picture of all glycaemic fluctuations including hyper- and hypoglycaemic episodes, which may be missed with fingerprick testing, were captured.’

**Methods
Variables**
Page 6, Lines 104–109: The justification for including specific glycaemic variability metrics is solid. However, the rationale for prioritizing SD and MAG over others in multivariate analysis remains unclear.

These were the two metrics that were significant in the univariate analysis so carried forwards into the multivariate analysis.

**Statistical Methods**
Page 7, Lines 135–145: The statistical approach is broadly appropriate, but the cutoff for including variables in multivariate analysis (p < 0.1) could lead to overfitting given the small sample size. Provide more details on how collinearity among predictors was assessed.

There are two related issues here, which, hopefully, we have clarfied in our revised manuscript. To take collinearity first, amomg the 8 variables that related to change from independent to dependent at p<0.1 that were included in our multivariable logistic regression, the two measures of glycaemic variability, SD and MAGE, were very highly correlated with a coefficient of 0.93 and variance inflation factors >7. In contrast, the other 6 variables showed no higher intercorrelation than 0.51 and variance inflation factors no higher than 2. We should, therefore, in the final multivariable logistic analysis have included two separate analyses, one that included only SD as a measure of glycaemic variability and one that included only MAGE. We have now carried out these separate analyses and found that the independent association with NIHSS on admission remains entirely unaffected and no other associations appear as significant. This is now reported as a footnote to Table 3 and in the Results section text (lines 192-195) of our revised manuscript.

The second Issue relates to weakness in the power of our analysis, which was referred to in our original Discussion in relation to the RI and DD between-group comparisons. We have now extended that section (lines 325-329) with a mention of the number of variables included in the multivariable analysis in relation to the number of observations. This further confirms that our study must be regarded as only providing pilot-level information and an indication of the greater numbers of observations required.

**Results
Participants**
Page 8, Lines 147–153: Missing data from 10 participants due to CGM errors should be explored further. Were these missing data random, or could they bias the results?

The missing data were random – just when the CGM device did not connect – no likelihood of bias

**Descriptive Data**
Page 9, Lines 152–157: The division of participants into subgroups (RI, DD, RD) is clear but unbalanced. Consider discussing how this imbalance might impact subgroup analyses.

We agree that the subgroups are imbalanced and whilst we have attempted to adjust for this in the analyses do accept that it may impact our results and hence conclusions. I have strengthened the discussion to emphasis this with the following statement:

‘Although this was intended as a hypothesis generating study, a greater number of participants, with more equitable balance between the subgroups, would have made our findings more robust.’

**Outcome Data**
Page 10, Lines 173–185: The decision not to include RD in statistical comparisons with RI/DD is reasonable but limits interpretability. A sensitivity analysis incorporating RD could strengthen conclusions.

The clinical interest in the data we present here must be in the transitions detected, the better, hopefully, to identify factors that might influence adverse clinical outcomes. In practice, we identified three groups distinguished by their overall course, namely remained independent (RI), remained dependent (RD) and fransitioned from independent to dependent (DD). Therefore, in terms of dependency status, the most clinically interesting group was DD and the appropriate comparison group was RI, since both groups began independent and one transitioned to dependency whereas the other did not. The equivalent comparator for our RD group would have consisted of participants starting dependent and transitioning to independent but, perhaps unsurprisingly, no such group emerged. The RD group is then a group without an effective comparator, of interest primarily for clinical characterisation of the patient groups we distinguished.

We have, nevertheless, undertaken a repeat of our logistic regression analysis with the RD group included, which then generates a model of transition to dependency with as comparison group, all those who did not transition to dependency regardless of initial status. The great majority of the variables that had shown a significant association with DD in univariate analysis ceased to be significant at p<0.1. Only atrial fibrillation (p=0.06) and NIHSS (p=0.006) remained. On multivariable analysis only NIHSS remained signdificant at p=0.01, whether the analysis was undertaken with SD or with MAGE separately or together. The loss of some univariate significances is to be expected given that combining RI and RD creates a comparison group that includes participants from two very different patient groups, one relatively healthy and the other with more advanced clinical characteristics (table 2). To that extent, inclusion of RD patients will have blunted discrimination of significant associations by introducing inconsistent data for the initial condition with which transition was to be defined. For completeness, we have added a brief paragraph to the Results that acknowledges the characteristics of RD group (lines 197-204) but, overall, feel that giving the group any more prominence could be confusing.

**Main Results**
Page 11, Lines 193–198: The multivariate model's failure to show glycaemic variability as significant deserves deeper exploration. Were SD and MAGE highly correlated with NIHSS, thereby diminishing their impact?

Neither SD (r^2^ 0.02) nor MAGE (r^2^ <0.0001) were correlated with NIHSS.

**Discussion
Key Results**
Page 13, Lines 200–208: The emphasis on NIHSS as the primary predictor of functional outcome is appropriate, but the failure of glycaemic variability metrics should be contextualized against prior conflicting findings.

I have added the following sentence:

‘Additionally, given the conflicting findings of previous studies, it is possible that GV and stroke outcomes are not intrinsically related and that, when identified, variable GV metrics are biomarkers for other pathophysiological processes or pharmacological interventions caused by or a result of having had a stroke. A larger study would help to discern this.’

**Limitations**
The limitation regarding single-center recruitment is mentioned but underemphasized. Additionally, the lack of data on specific interventions (e.g., use of intravenous glucose) during monitoring introduces potential bias.

Thank you; I have added the following sentences:

‘The focus for this hypothesis-generating study was on GV however it may accrue, thus although we did not collect data on the use of glucose containing intravenous fluids use or other interventions, all participants with diabetes had regular fingerprick glucose testing and management of blood glucose was undertaken according in line with local guidelines and clinical assessment by the treating medical team.’

And:

‘The study recruited from a single *centre in which all participants had the benefit of consultant led multidisciplinary care in a dedicated hyperacute stroke unit*, which may limit wider interpretation of the results to other stroke units or hospitals with different practices.’

**Interpretation**
The authors miss an opportunity to discuss the clinical implications of their findings. How might these results influence the use of CGM in stroke patients or guide future trias?

Thank you for raising this – a very fair point. I have added this to the discussion:

‘As the use of CGM becomes more widespread in people with type 2 diabetes, and in inpatient settings, challenges we encountered in undertaking this study highlight potentially important clinical implications.^32,33^ In particular, the continued use of CGM technology in patients requiring MRI imaging will require additional training and support of both inpatient and imaging staff. The CGM used in this study has been shown to be safe in MRI scanners although national guidelines currently recommend their removal.^33,34^ Looking ahead, until this tension is resolved, local guidance and education will be required to enable and implement minimally interrupted use of CGM in people who require MRI scans.’

32.   Garg SK, Hirsch IB, Repetto E, et al. Impact of continuous glucose monitoring on hospitalizations and glucose control in people with type 2 diabetes: real-world analysis. Diabetes Obes Metab 2024; 26(11): 5202-5210 doi:10.1111/dom.15866

33.   Avari P, Choudhary P, Lumb A, et al. Using technology to support diabetes care in hospital: Guidelines from the Joint British Diabetes Societies for Inpatient Care (JBDS-IP) group and Diabetes Technology Network (DTN) UK. Diabet Med 2024; 00:e15452 doi:10.1111/dme.15452

34.   Thomas C, Welsh JB, Lu S, et al. Safety and Functional Integrity of Continuous Glucose Monitoring Components After Simulated Radiologic Procedures. J Diabetes Sci Technol 2021;15(4):781-785 doi: 10.1177/1932296820920948

**Supplementary Information**
The inclusion of post hoc power calculations is commendable. However, presenting this information graphically could aid reader comprehension.

The post-hoc power calculations we present at the end of our Results section were really intended to illustrate the extent to which the numbers we recruited approached those needed for a fully powered study, rather than support for any recommendations as to how others should design their studies. The summary data we present and used for estimating numbers needed could be used by other investigators for powering their own studies, which could involve any one of many different combinations of anticipated study group size differences and anticipated group differences. In view of this, we are unsure as to how any graphic presentation of numbers needed might help. We could present numbers as a table but, again, numbers needed are so dependent on study design, we feel that even that would give the impression of over-determination as to how others should proceed.

**Minor Issues**
Formatting inconsistencies in tables (e.g., Table 2) detract from readability. Ensure consistent decimal places for all variables.

Done

Some abbreviations (e.g., MAG, TIR) are not defined upon first use in the main text.

These are defined in this paragraph in the Methods under Study Protocol:

Glucose data downloaded from receiver after sensor removal. Data within first 72 hours after admission were used to calculate mean glucose, percentage (%) time in range (TIR; 70–180 mg/dL), % time below range (TBR; <70 mg/dL and <54mg/dL), % time above range (TBR; >180 mg/dL) and GV including standard deviation (SD), % coefficient of variation (CV), mean absolute glucose (MAG), mean amplitude of glucose excursions (MAGE), continuous overlapping net glycaemic action (CONGA) and low blood glucose index (LBGI).
